# Genome Editing *VEGFA* Prevents Corneal Neovascularization In Vivo

**DOI:** 10.1002/advs.202401710

**Published:** 2024-04-06

**Authors:** Zhenhai Zeng, Siheng Li, Xiuhong Ye, Yiran Wang, Qinmei Wang, Zhongxing Chen, Ziqian Wang, Jun Zhang, Qing Wang, Lu Chen, Shuangzhe Zhang, Zhilin Zou, Meimin Lin, Xinyi Chen, Guoli Zhao, Colm McAlinden, Hetian Lei, Xingtao Zhou, Jinhai Huang

**Affiliations:** ^1^ Eye Institute and Department of Ophthalmology Eye & ENT Hospital Fudan University Key Laboratory of Myopia Chinese Academy of Medical Sciences Shanghai 200000 China; ^2^ Shanghai Key Laboratory of Visual Impairment and Restoration Shanghai 200000 China; ^3^ School of Ophthalmology and Optometry and Eye Hospital Wenzhou Medical University Wenzhou Zhejiang 325000 China; ^4^ Key Laboratory for Regenerative Medicine Ministry of Education Jinan University Guangzhou 510000 China; ^5^ Department of Ophthalmology 2nd Affiliated Hospital of Nanchang University Nanchang 330000 China; ^6^ Corneo Plastic Unit & Eye Bank Queen Victoria Hospital East Grinstead RH19 3AX UK; ^7^ Shenzhen Eye Hospital Shenzhen Eye Institute Jinan University Shenzhen 518000 China

**Keywords:** (CRISPR)/Cas9, CNV, genome editing, VEGFA

## Abstract

Corneal neovascularization (CNV) is a common clinical finding seen in a range of eye diseases. Current therapeutic approaches to treat corneal angiogenesis, in which vascular endothelial growth factor (VEGF) A plays a central role, can cause a variety of adverse side effects. The technology of Clustered Regularly Interspaced Short Palindromic Repeats (CRISPR)/Cas9 can edit VEGFA gene to suppress its expression. CRISPR offers a novel opportunity to treat CNV. This study shows that depletion of VEGFA with a novel CRISPR/Cas9 system inhibits proliferation, migration, and tube formation of human umbilical vein endothelial cells (HUVECs) in vitro. Importantly, subconjunctival injection of this dual AAV‐SpCas9/sgRNA‐*VEGFA* system is demonstrated which blocks suture‐induced expression of VEGFA, CD31, and α‐smooth muscle actin as well as corneal neovascularization in mice. This study has established a strong foundation for the treatment of corneal neovascularization via a gene editing approach for the first time.

## Graphical abstract

Corneal neovascularization (CNV) is a common clinical finding observed in various eye diseases, which can lead to blindness. Using the gene editing technology CRISPR/Cas9, sgRNA is selected to regulate the *VEGFA* gene expression that promotes neovascularization, thereby achieving therapeutic purposes. This study establishes a solid foundation for the treatment of CNV through a gene editing approach for the first time.

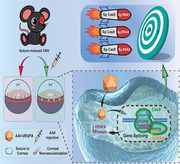

## 1 Introduction

The incidence of corneal neovascularization (CNV) is ≈1.4 million per year.^[^ ^1^ ^]^ The cornea is an important refractive structure of the visual system, and the avascularity of the cornea is important to keep the cornea transparent.^[^ ^2^ ^]^ Under normal circumstances, the cornea is an immune‐privileged organ without blood and lymphatic vessels.^[^ ^3^ ^]^ However, under the action of pathogenic factors such as infection, allergy, trauma, immune rejection, ischemia, hypoxia, and chemical damage, the limbal vascular network forms new capillaries.^[^ ^4^ ^]^ Neovascular invades the cornea, causing turbidity of the corneal stroma, irregularities of the corneal surface, corneal scarring and edema, lipid deposition, obstruction of light, and loss of vision.^[^ ^5^ ^]^ The antiangiogenic factors and pro‐angiogenic factors of a normal healthy cornea maintain a stable balance.^[^ ^6^ ^]^ Once the factors that promote angiogenesis are stronger than those that inhibit angiogenesis, the cornea will produce pathological neovascularization.^[^ ^7^ ^]^ Vascular endothelial growth factor (VEGF) A is a vital angiogenic factor,^[^ ^8^ ^]^ and the up‐regulation of VEGFA is the most important cause of corneal neovascularization.^[^ ^9^ ^]^ Hence, VEGFA is a therapeutic target in corneal neovascularization.^[^ ^10^ ^]^ However, traditional anti‐VEGFA therapeutic drugs such as small molecule inhibitors and anti‐VEGFA antibodies have limited effects, are unstable, have a short duration of action, and may induce adverse reactions.^[^ ^11^ ^]^ Therefore, the treatment of corneal angiogenesis remains a major challenge.

The technology of Clustered Regularly Interspaced Short Palindromic Repeats (CRISPR)/Cas9 offers novel opportunities to treat human diseases.^[^ ^12^ ^]^ CRISPR/Cas9 technology has been used to treat laser‐induced choroidal neovascularization in mice; a model of age‐related macular degeneration.^[^ ^13^ ^]^ Here, we hypothesized that gene editing with CRISPR/Cas9 could be used to suppress VEGFA expression to inhibit corneal neovascularization in mice.

## 2 Results and Discussion

### 2.1 Suppression of VEGFA Expression by CRISPR/Cas9 In Vitro

**Figure 1:**
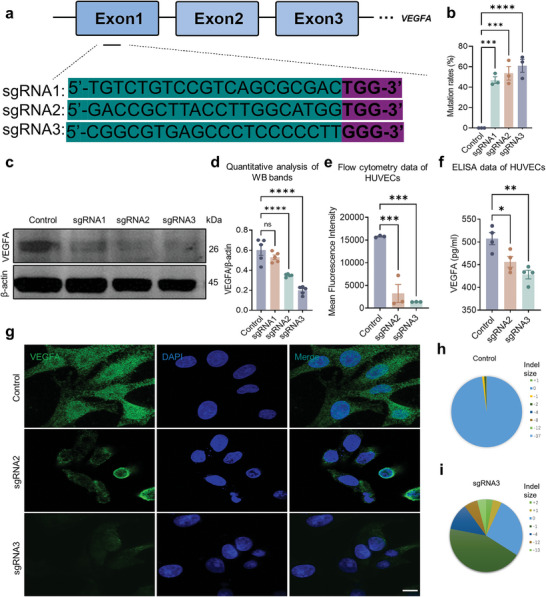
Selection of the best one among 3 sgRNA for editing genomic *VEGFA*. a) Schematic diagram of CRISPR/Cas9 sgRNA design. The black font indicates the sgRNA sequence, and the red one indicates the PAM. b) Quantification of the editing efficiency in Figure S1a–c (Supporting Information). Dots indicate individual values and bar graphs represent the mean ± standard deviation (SD), *n* = 3. c) Western blotting analysis of VEGFA expression in lysates of HUVECs transfected with CRISPR/Cas9 plasmids. d) Quantitative analysis of western blotting bands. e) Analysis of VEGFA expression by flow cytometry in HUVECs transfected with CRISPR/Cas9 plasmids. f) ELISA analysis of VEGFA in the culture medium of HUVECs transfected with CRISPR/Cas9 plasmids. g) Immunofluorescence analysis of VEGFA expression in HUVECs transfected with CRISPR/Cas9 plasmids. Scale bar, 20 µm. h,i) The next generation sequencing (NGS) of SpCas9/sgRNA3 and Control. Dots indicate individual values and bar graphs represent the mean ± SD, *n* ≥ 3.

To design sgRNAs for guiding SpCas9 to edit genomic VEGFA, an online tool CHOPCHOP was used to select three sgRNAs from genomic loci of both mouse and human *VEGFA* genes,^[^ ^14^ ^]^ which were correspondingly referred to as sgRNA1, −2, and −3 as shown in **Figure** **1**a. Subsequently, a T7E1 assay was utilized to test their editing efficiency in human embryonic kidney (HEK) 293T cells. As shown in Figure 1b and Figure S1a–c (Supporting Information), the results demonstrated that these three sgRNAs directed SpCas9 to edit genomic *VEGFA*. In addition, Sanger sequencing results revealed that there were multiple peaks at the 5’ end of sgRNA targeting position of genomic *VEGFA* locus after transfection with SpCas9/sgRNA1‐3 (Figure S2a–c, Supporting Information), indicating that nonhomologous end joining (NHEJ) was engaged by SpCas9/sgRNA1‐3, mixed population of indels (and wild type) of *VEGFA* was present. We used Cas‐OFFinder, a fast and versatile algorithm to search for potential off‐target sites of Cas9 RNA‐guided endonucleases. T7E1 assay indicated that all the potential off‐target sites were not cleaved by SpCas9/sgRNA1‐3(Figure S1d,e, Supporting Information).

We next transfected SpCas9‐sgRNA1, SpCas9‐sgRNA2, and SpCas9‐sgRNA3 into HUVECs and analyzed them with western blot. Notably, sgRNA1 showed the lowest efficiency among the three sgRNAs (Figure 1c,d). Therefore, we eliminated SpCas9‐sgRNA1 and transfected SpCas9‐sgRNA2 and SpCas9‐sgRNA3 into HUVECs in the subsequent experiments. The flow cytometry showed that there was a significant reduction in VEGFA in HUVECs transduced with SpCas9‐sgRNA2 and SpCas9‐sgRNA3 (Figure 1e); in addition, ELISA and immunofluorescence results also demonstrated that there was less VEGFA in HUVECs transduced with SpCas9‐sgRNA2 and SpCas9‐sgRNA3 than those with control plasmids (Figure 1f,g). The data of the next‐generation sequencing (NGS) reveal that over 70% of *VEGFA* DNA was cleaved by SpCas9/sgRNA3 (Figure 1h,i). All the data above indicated that SpCas9‐sgRNA3 inhibited the expression of VEGFA protein better than SpCas9‐sgRNA2. Thus, we selected SpCas9‐sgRNA3 for subsequent experiments.

### 2.2 Editing VEGF Attenuates Proliferation, Migration, and Tube Formation of Cultured Human Vascular Endothelial Cells

**Figure 2:**
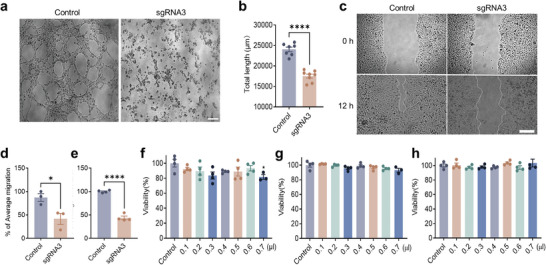
Editing *VEGFA* inhibited angiogenesis in vitro. a,b) Inhibition of HUVECs’ tube formation by SpCas9‐sgRNA3. Scale bar, 50 µm. c,d) Prevention of HUVECs’ migration by SpCas9‐sgRNA3. Scale bar, 100 µm. e) Suppression of HUVECs’ proliferation by SpCas9‐sgRNA3 with a CCK8 assay. f–h) Attenuation of HUVECs’ viability by SpCas9‐sgRNA3 with a CCK8 assay at time points of 24, 48, and 72 h. Dots indicate individual values and bars represent the mean ± SD, *n* ≥ 4.

There is an autocrine loop of VEGFA/VEGFR signaling in vascular endothelial cells and interruption of this loop impacts cellular functions including proliferation, migration, and tube formation.^[^ ^15^ ^]^ As a consequence, we found that interference of VEGFA production in HUVECs transduced with the SpCas9‐sgRNA3 inhibited tube formation of these endothelial cells (**Figure** **2**a,b), and their migration examined by a scratch wound‐healing assay (Figure 2c,d). In addition, a CCK8 assay also showed that there were less proliferative cells transfected with SpCas9‐sgRNA3 than control (Figure 2e).

To examine whether the toxicity of the transfection reagent may cause false positive results, we set up lipo3000 with gradient solubility and detected the viability of HUVECs cells at 24, 48, and 72 h. The results showed that there was no significant difference in HUVECs transfected with the SpCas9‐sgRNA3 compared with control (Figure 2f–h), suggesting the SpCas9‐sgRNA3 targeting *VEGFA* could inhibit neovascularization.

### 2.3 Editing VEGFA Prevents Suture‐Induced Corneal Neovascularization

At first, we tested whether the SpCas9‐sgRNA3 could work in mouse cells. Thereby, SpCas9‐sgRNA3 was transfected into a mouse cell line (RAW264.7). As shown in Figure S3a–d (Supporting Information), there was a significant decrease in VEGFA in the supernatant of cultured RAW264.7 cells and their cell lysates, suggesting the SpCas9‐sgRNA3 could effectively result in editing VEGFA in mice.

**Figure 3:**
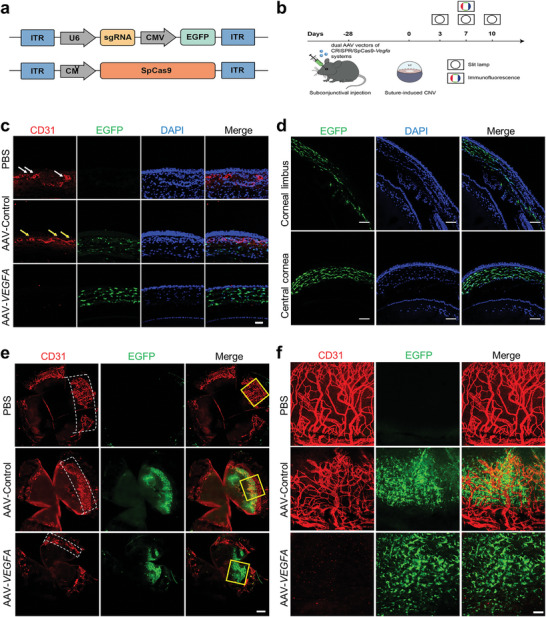
Editing *VEGFA* with AAV‐CRISPR/Cas9 prevented corneal angiogenesis in vivo. a) Schematic of dual AAV vectors of CRISPR/SpCas9‐*VEGFA* system. b) Schematic overview of the in vivo studies using the dual AAV vectors of CRISPR/SpCas9‐*VEGFA* system. c) Immunofluorescence staining corneal cryosections with an antibody against CD31 antibody. The white and yellow arrows point to the corneal neovascular ring lumen. AAV‐*VEGFA*: AAV‐SpCas9‐sgRNA‐*VEGFA*. Scale bar, 50 µm. d) Evaluation of the central cornea and peripheral cornea infected by the AAV‐ SpCas9‐sgRNA‐*VEGFA* system. Scale bar: 100 µm. e) Whole cornea was immunofluorescence stained with indicated antibodies, the white dashed boxes mark the area of corneal neovascularization. Scale bar: 300 µm. f) Partially enlarged images of the corneal infected by the dual AAV vectors of SpCas9‐sgRNA‐*VEGFA* system in the yellow frames of Figure 3e. Scale bar: 50 µm.

To this end, dual AAVs of AAV‐SpCas9 and AAV‐sgRNA3 targeting mouse VEGFA (1:1, 2.5 × 10^9 ^VG) were planned to be injected subconjunctivally under the bulbar conjunctiva of mice (**Figure** **3**a,b) immediately posterior to the limbus. A corneal trephine was utilized to demarcate a 2 mm diameter circle centered around the pupil, upon which corneal neovascularization induced by sutures was performed (Figure S4a–c, Supporting Information). After the injection for 28 days, surgical placement of a suture was performed to induce corneal neovascularization, which would appear on day 3 after this surgery and reach the peak on day 10 (Figure 3b). The results showed that there was EGFP expression in both the central cornea and corneal limbus (Figure 3c,d, Figure S5a,b, Supporting Information). Intrastromal injection and subconjunctival injection are two commonly employed methods for corneal administration. Comparative analysis revealed that subconjunctival injection resulted in reduced corneal injury compared to intrastromal injection, with a significantly lower risk of corneal epithelial defect occurrence (Figure S5c, Supporting Information). The corneal fluorescein staining score demonstrated a notably diminished score following subconjunctival injection as opposed to corneal stromal injection (Figure S5d, Supporting Information). These findings suggest that the subconjunctival injection of the dual AAVs targeting *VEGFA* system not only enables efficient delivery but also exhibits favorable biosecurity.

Additionally, there was some cornea tissue positively stained with CD31, a marker of vascular endothelial cells (Figure 3c), demonstrating the sutures stimulated neovascularization in the mouse cornea. Notably, there was much less neovascularization in the corneas injected with the dual AAVs of AAV‐SpCas9 and AAV‐sgRNA‐*VEGFA* compared with those injected with controls, indicating editing *VEGFA* inhibits suture‐induced corneal neovascularization in mice (Figure 3c).

**Figure 4:**
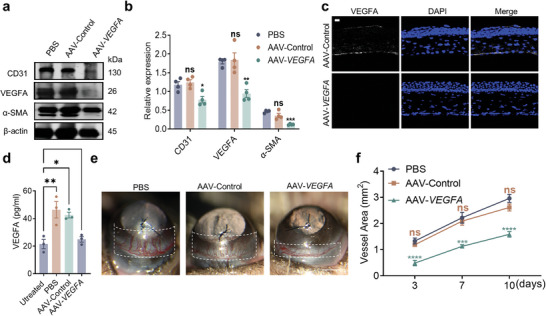
Editing *VEGFA* using the AAV‐SpCas9/sgRNA‐*VEGFA* system attenuated Suture‐induced corneal angiogenesis and expression of α‐smooth muscle action *(α‐SMA)* in vivo. a) Western blotting analysis of corneal tissues infected with AAV‐SpCas9‐sgRNA‐*VEGFA* using indicated antibodies. b) Quantitative analysis of western blotting bands in A. c) Confocal images of VEGFA expression in the cornea tissue transduced with AAV‐SpCas9/AAV‐sgRNA‐*VEGFA*. Scale bar, 20 µm. d) VEGFA expression evaluated by ELISA in corneal infected with the dual AAVs. e) Suture‐induced corneal neovascularization was attenuated by the dual AAVs. The white dashed box marks the range of pathological corneal neovascularization. f) Quantitative analysis of pathological corneal neovascularization. Dots represent individual values and bar graphs represent the mean ± SD, *n* ≥ 3.

In order to clearly assess the distribution of neovascularization, we unfolded the cornea. The results showed that there was significantly more neovascularization around the cornea in the control eyes than in the eyes receiving the dual AAVs targeting *VEGFA* (Figure 3e,f). Furthermore, western blotting results showed that there were less VEGFA, CD31, and α‐SMA in the corneas injected with the dual AAVs targeting *VEGFA* than the controls (**Figure** **4**a,b). Immunofluorescence and ELISA analysis also showed that there was less VEGFA in corneal tissue injected with dual AAVs targeting *VEGFA* than with controls (Figure 4c,d).

Corneal neovascularization was observed by slit lamp examination on the 7th day after suturing. In the PBS‐injected group, suture‐induced neovascularization as shown in the white dashed box in Figure 4e, and injection of control AAVs did not impact corneal neovascularization, whereas injection of dual AAVs targeting *VEGFA* inhibited suture‐induced neovascularization (Figure 4e). There was less corneal neovascularization on days 3, 7, and 10 after suturing the corneas injected with the dual AAVs targeting *VEGFA* (Figure 4f). In order to compare the effects of dual AAVs targeting *VEGFA* on CNV with Conbercept, the commonly used recombinant anti‐VEGF medication, we evaluated their respective inhibitory capacities at an equivalent viral dose. Anterior segment photography revealed that while Conbercept exhibited some inhibition of corneal neovascularization compared to the control group, the dual AAVs targeting *VEGFA* demonstrated a superior inhibitory effect 5 days after suturing the corneas (Figure S6a, Supporting Information). Specifically, Conbercept reduced neovascularization by 63.33% ± 8.62%, whereas the dual AAVs of AAV‐SpCas9 and AAV‐*VEGFA* achieved a reduction of 72.30% ± 2.30% (Figure S6b, Supporting Information). Neovascular immunofluorescence staining of the expanded cornea revealed that both the dual AAVs targeting the *VEGFA* system and Conbercept effectively suppressed corneal neovascular growth, with the dual AAVs targeting *VEGFA* system exhibiting superior inhibitory efficacy compared to Conbercept (Figure S6c, Supporting Information). Together, these results demonstrate that the dual AAVs of AAV‐SpCas9 and AAV‐*VEGFA* are able to effectively inhibit neovascularization.

### 2.4 Subconjunctival Injection of Dual AAVs Targeting *VEGFA* does not Interfere with Retinal Function and has Excellent Biosecurity

**Figure 5:**
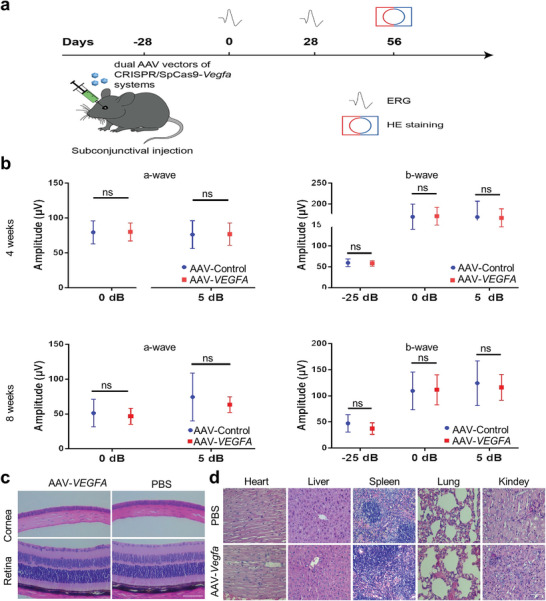
The safety assessment of dual AAV vectors of CRISPR/SpCas9‐*VEGFA* system. a) Schematic overview of the safety assessment of AAV‐SpCas9 together with AAV‐sgRNA‐*VEGFA* in vivo. b) ERG evaluation of retinal function in mice injected with the dual vectors of AAV‐SpCas9 and AAV‐sgRNA‐*VEGFA* for 4 and 8 weeks. c,d) Hematoxylin and Eosin staining of the cornea, retina, heart, liver, spleen, lung, and kidney of mice injected with the dual vectors of AAV‐SpCas9 and AAV‐sgRNA‐*VEGFA* system did not affect the normal physiological construction of the cornea and retina. Scale bar: 200 µm in D, and 100 µm in E.

We investigated whether suppression of VEGFA expression in ocular tissues with CRISPR/Cas9 affects retinal function. We examined retinal function with an electroretinogram (ERG) and retinal structure with H&E staining after AAV‐CRISPR/Cas9 injection (**Figure** **5**a). The results showed that there was no significant difference in ERG a and b waves in eyes injected with dual AAVs targeting *VEGFA* compared to controls for 4 or 8 weeks (Figure 5b).

We next assessed whether the dual AAVs could affect other organs after subconjunctival injection. Thereby, we examined frozen sections of the retina, conjunctiva, iris, trabecular meshwork, extraocular muscle, optic nerve, ciliary body, heart, liver, spleen, lung, and kidney with confocal microscopy. Results showed that while there was an EGFP signal in the cornea and conjunctiva, there was no such signal found in all other examined organs (Figures S7 and S8, Supporting Information), suggesting that the subconjunctival injection of the dual AAVs may only transduce the corneal and conjunctiva tissues. Subsequently, we also evaluated the morphology of the cornea, retina, heart, liver, spleen, lung, and kidney. The results showed that there was no significant change in these examined tissues after subconjunctival injection of the dual AAVs targeting VEGF compared to controls (Figure 5c,d). The T helper cell 17 (Th17), an identified subset of T cells that secrete interleukin 17 (IL‐17), plays a crucial role in the immune response against viral vector invasion.^[^ ^16^ ^]^ In the quiescent state, Th0's capacity to differentiate into Th17 is limited, resulting in only a minimal population of Th17 cells within the spleen (Figure S9a, Supporting Information). However, upon PMA/Ionomycin stimulation in the positive control group, there was a significant increase in the number of Th17 cells within the spleen (Figure S9b, Supporting Information). Interestingly, 7 days after subconjunctival injection of the dual AAVs targeting *VEGFA* system, no significant alteration was observed in the number of splenic Th17 cells (Figure S9c, Supporting Information). All these data together indicate that there is no toxicity detectable after subconjunctival injection of the dual AAVs targeting *VEGFA*.

## 3 Conclusion

Current treatments of corneal neovascularization include topical corticosteroids and nonsteroidal anti‐inflammatory drugs, photodynamic therapy, laser photocoagulation, fine‐needle diathermy, and amniotic membrane transplantation.^[^ ^5^ ^]^ However, these treatments have limited clinical efficacy, and some eyes fail to respond to traditional treatment or have even worsened after treatments.^[^ ^17^ ^]^ Corticosteroids are associated with raised intraocular pressure and posterior subcapsular cataract,^[^ ^18^ ^]^ and laser photocoagulation treatment is prone to recurrence.^[^ ^11^ ^]^ Due to the instability of protein and RNA, repeated treatments are often necessary.^[^ ^19^ ^]^

Most cases of hypoxic corneal neovascularization are the result of contact lens use.^[^ ^20^ ^]^ Under hypoxic conditions, VEGFA is upregulated in corneal epithelial cells in an attempt to increase the oxygen supply to the cornea.^[^ ^21^ ^]^ Pathological ocular angiogenesis is regulated by a variety of angiogenic factors, including VEGFs,^[^ ^22^ ^]^ platelet‐derived growth factors (PDGFs), fibroblast growth factors (FGFs), insulin‐like growth factors (IGFs), transforming growth factors‐β (TGFβs), endothelin, galectin, and integrin.^[^ ^23^ ^]^ Among them, VEGFA is considered to be the most effective pro‐angiogenic factor, and a traditional treatment strategy is to neutralize VEGFA.

The emergence of CRISPR gene editing technology has allowed us to regulate the expression level of VEGFA. As long as the editing event occurs, resulting in a frameshift mutation in the *VEGFA* gene, the cell cannot transcribe the biologically active VEGFA protein, so the therapeutic effect can be maintained for a long period. This new treatment strategy has a high potential to solve the shortcomings of repeated treatments and unobvious effects caused by traditional treatments. Due to its simple working principle, strong operability, and low cost, the CRISPR/Cas9 system is a revolutionary innovation in the treatment of many diseases.^[^ ^24^ ^]^

The CRISPR/Cas9 system has been used to study the role of specific genes at the cellular level and in living animals in the research of ophthalmic diseases.^[^ ^25^ ^]^ For example, in a first phase I/II clinical trial approved by the United States Food and Drug Administration (FDA) in 2017, the CRISPR/Cas9 system was used to treat type 10 Leber congenital amaurosis (LCA), which is a congenital retinal dystrophy. In the past few decades, gene therapy has mainly been focused on small interfering RNA silencing *VEGFA* gene to treat neovascular eye disease.^[^ ^26^ ^]^ Since the rapid development of gene editing technology, there are now more studies using the CRISPR/Cas9 system to knock out the *VEGFA* gene to treat age‐related macular degeneration, diabetic maculopathy/retinopathy, and other retinal diseases in preclinical studies.^[^ ^27^ ^]^ These studies have implied the feasibility and safety of the CRISPR/Cas9 system for depleting VEGFA expression. Therefore, in this study, we leveraged the CRISPR/Cas9 system to knock out the *VEGFA* gene for the treatment of suture‐induced corneal neovascularization in mice. To the best of our knowledge, this is the first report to employ the AAV‐mediated CRISPR/Cas9 system for treating corneal neovascularization in vivo, shedding light on treating such an eye disease with the CRISPR strategy.

## 4 Experimental Section

### Mice

Four‐week‐old mice (C57BL/6J, male) without ocular disease were selected for the animal experiment. All animal experiments were approved by the EYE & ENT Hospital of Fudan University's Animal Ethics Committee, Shanghai, China (IRBEENT‐20220210a). All the mice were placed in an animal house with 12 h of light/darkness and provided with food and water.

### Major Reagents

VEGFA antibody (1:1000 for western blot, AB1876‐I) was purchased from Merck Millipore (St. Louis, MO USA). α‐SMA antibody (1:1000 for western blot, 19245S), CD31 (1:1000 for western blot, 1:500 for fluorescence immunoassay, 77699S), and β‐actin (4970S, 1:1000 for western blot) were purchased from Cell Signaling Technology (Danvers, MA). IRDye 800CW Donkey anti‐Rabbit IgG Secondary Antibody (1:20 000 for western blot, 926–32213) and IRDye 680RD Donkey anti‐Rabbit IgG Secondary Antibody (1:20 000 for western blot, 926–68073) were purchased from LI‐COR Biotechnology (Lincoln, Nebraska USA). Enhanced chemiluminescent substrate for detection of HRP was purchased from Thermo Fisher Scientific (Waltham, MA, USA). Opti‐MEM Reduced Serum Medium was purchased from Gibco (Thermo Fisher Scientific, Waltham, MA, USA). Lipofectamine 3000 was purchased from Thermo Fisher Scientific (Waltham, MA, USA).

### Materials

Cell counting kit‐8 (CCK‐8) and BCA assay kit were purchased from Beyotime (Shanghai, China). The bioagents for cell culture were purchased from Gibco (Invitrogen, USA). Milli‐Q grade water (Millipore, Bedford, MA) was used for the preparation of the solution and mobile phase.

### Cell Culture

The primary human umbilical vein endothelial cells (HUVECs) were purchased from CHI SCIENTIFIC Inc. HUVECs were cultured in endothelial culture medium (ScienCell, USA) supplemented with 10% fetal bovine serum (FBS, Gibco) and endothelial growth factor (ScienCell, USA). The mouse mononuclear macrophage cells (RAW264.7) and the Human Embryonic Kidney 293T cells (HEK 293T cells) were purchased from Shanghai Chinese Academy of Sciences. HEK‐293T cells were cultured in high glucose DMEM medium supplemented with 10% fetal bovine serum (FBS, Gibco). RAW264.7 cells were cultured in low glucose DMEM medium supplemented with 10% fetal bovine serum (FBS, Gibco). All cells were cultured at 37 °C in a humidified 5% CO_2_ atmosphere.

### Transduction of Cultured Cells

The cells were cultured in a 24‐well plate. Lipofectamine 3000 and SpCas9 plasmids (Lipofectamine 3000 2 µL per well, plasmids 1 µg per well) were thoroughly mixed in the Opti‐MEM, while the control group only had Opti‐MEM and Lipofectamine 3000 added. After 6 h of incubation, there was a change to a fresh medium. Two days later, the cells were photographed under an immunofluorescence microscope to determine the Lipofectamine 3000 transduction efficiency. Then, the cells were lysed with 1× sample buffer for western blotting analysis or collected for genomic DNA isolation.

### Cell Proliferation

HUVECs were seeded in a 96‐well plate at a density of 1 × 10^4^ cells per well and incubated overnight in a CO_2_ incubator at 37 °C. Subsequently, as described earlier, the SpCas9 plasmids were transfected into the cells using Lipofectamine 3000. After 72 h of incubation, 10 µL CCK8 solution was added to each well for an additional incubation of 2 h, and the absorbance values were measured using a microplate reader (Bio‐Rad Laboratories, Hercules, CA, USA) at 450 nm. The cell viability was calculated using the following equation:

(1) $$\mathrm{Cell}\;\mathrm{viability}\left(\%\right)\;=\;\mathrm{absorbance}\;\mathrm{test}/\mathrm{absorbance}\;\mathrm{control}$$

### Wound‐Healing Assay

The wound‐healing migration assay was performed as described. Briefly, when the HUVECs reached 70% confluence in 24‐well plates, the SpCas9 plasmids were transfected with Lipofectamine 3000. Then cell monolayer was scraped with a sterile pipette tip (200 µL). The cells were washed twice to remove detached cells. The physical gap was photographed at 0 and 12 h under a microscope. Quantification was performed by measuring the number of pixels in the wound area, using Adobe Photoshop (Adobe Systems, San Jose, CA, USA) and analyzed by using Image J software. The cell migration rates were calculated with the following formula:

(2) m=(r−n)/r×100%

where *m* is the migration, *n* is the area of scratch at 12 h, and *r* is the initial area of scratch.

### Tube Formation Assay

Tube Formation assay was used to evaluate the effect on tube formation when the *VEGFA* gene was knocked out by SpCas9. Briefly, Cultrex Basement Extract (BME) (Trevigen, Gaithers burg, MD, USA) from storage at −80 °C was thawed overnight on ice. Then a 96‐well plate was placed on ice for at least 10 to 15 min, and the solution of BME (60 µL) was transferred into each well. This plate was subsequently incubated at 37 °C for 30–60 min to polymerize the gel. After 1 h, HUVECs cells were transfected with Lipofectamine 3000 and SpCas9 plasmids at a density of 2 × 10^4^ per well in 100 µL of culture medium and were plated on top of each polymerized BME gel. Images of the tubes were photographed at 1–6 h post assay under a light microscope. The data were imported as a TIFF file into Image J software for calculating the total length of tubes, using an angiogenesis analysis module. The data from three independent experiments were analyzed with Prism 8 software (GraphPad Software, Inc., La Jolla, CA, USA).

### Genomic DNA Isolation and PCR

Genomic DNA was isolated from 293T cells after transfection with the CRISPR/Cas9 systems using the FastPure Blood/Cell/Tissue/Bacteria DNA Isolation Mini Kit (Vazyme Biotech Co. Ltd, Nanjing, China, # DC112‐01) following the manufacturer's protocol. The target sites were PCR‐amplified and purified with a Gel DNA Extraction Kit (Vazyme Biotech Co. Ltd, Nanjing, China, DC301) for sequencing.

### Western Blot

Cells or corneal tissue homogenate were lysed in RIPA 1× sample buffer, which was diluted with an extraction buffer (10 mm Tris‐HCl, pH 7.4, 5 mm EDTA, 50 mm NaCl, 50 mm NaF, 1% Triton X‐100, 20 µg mL^−1^ aprotinin, 2 mm Na_3_VO_4_, and 1 mm phenylmethylsulfonyl fluoride) from the 5× protein sample buffer (25 mm EDTA (pH = 7.0), 10% sodium dodecyl sulfate (SDS), 500 mm dithiothreitol, 50% sucrose, 500 mm Tris‐HCl (pH = 6.8), and 0.5% bromophenol blue). The lysates were boiled for 5 min and then centrifuged for 10 min at 12 000× g. Proteins from the samples were separated by 10% SDS‐PAGE, transferred to polyvinylidene difluoride membranes, and subjected to western blot analysis. Experiments were repeated at least three times. Signal intensity was determined by densitometry using NIH Image J software.

### A Mouse Model of Corneal Neovascularization

Establishment and medication of suture‐induced corneal neovascularization (CNV): the CNV model induced by suture was established to evaluate the efficacy of AAV2/9‐CRISPR/SpCas9‐*VEGFA* systems in vivo. After systemic anesthesia with isoflurane (RWD Inc) and topical anesthesia with proparacaine (Alcon, Fort Worth, TX, USA), mice were placed in a lateral position on standby. Utilize a 2 mm diameter corneal trephine to delineate a circular marking, precisely centered on the pupil within the central region of the cornea. The circular filter paper with a diameter of 2 mm was pasted on the center of the cornea, and the edge of the filter paper was used to determine the location of the needle insertion. Only the right eye was sutured on each mouse; once on the upper temporal area of the cornea. All mice were randomly grouped and subconjunctivally injected around the stitches with a total 5 µL solution: 1) PBS, 2) AAV‐control, or 3) AAV2/9‐CRISPR/SpCas9‐*VEGFA* systems. The CNV condition was observed by slit lamp biomicroscopy (Kanghua, Chongqing) with a flashlight to evaluate anti‐CNV efficacy on days 3, 7, and 10. The CNV length and area were determined using Image Pro Plus 6 and calculated according to a previous fan‐shaped area method. The equation is shown below:

(3) $$S\;=\;C\;÷\;12\;\times \;\pi \;\times \;[r^{2\;}-\;{\left(r\;-\;L\right)}^{2}]$$

*S* is the area of CNV, *C* is clock hours of fan‐shape CNV, *r* is corneal radius, *L* is the average length of new blood vessel at the beginning of limbus.

### Immunofluorescence Observation

The right eyes of mice were carefully harvested after euthanasia and promptly frozen by liquid nitrogen in optimal cutting temperature (OCT) compound for frozen section. The tissues were frozen‐sectioned (10 µm) and fixed in 4% paraformaldehyde for 15 min, then they were rinsed by PBS for three times. The nonspecific binding site of section was blocked with ready‐to‐use goat serum (Boster) at room temperature for 1 h before adding rabbit anti‐CD31 antibody (1:500) at 4 °C overnight. After being washed with PBST (PBS with 0.05% Tween) for three times, goat anti‐rabbit Alexa Fluor 594 (1:500) were added into the sections for 2 h incubation at room temperature in the dark box, and the fluorescence signal of tissues was recorded by confocal fluorescence microscopy (Zeiss LSM880).

### Ocular Biocompatibility Assay

The ocular safety of AAV‐CRISPR/SpCas9‐*VEGFA* systems via the sub‐conjunctival injection route was evaluated by electroretinography (ERG, RETI‐Port) and histopathological observation. The healthy male mice were selected and anesthetized systemically and topically. The right eye of a mouse was subconjunctivally injected in the temporal quadrant with 5 µL AAV‐CRISPR/SpCas9‐*VEGFA* systems, while the left eye was treated with an equal volume of PBS as a control. The visual function was assessed using a RETI port system of ERG 4‐ and 8‐week post‐injection. After dark adaption overnight, mice were anesthetized with 5% (w/v) chloral hydrate (0.08 mL per 10 g) by intraperitoneal injection.

Then the pupil was dilated with tropicamide, topical ocular surface was anesthetized with proparacaine and humidified with 0.1% sodium hyaluronate (Santen, Japan) for better electric conduction. Subsequently, a ground pin electrode was inserted subcutaneously in the tail and two reference pin electrodes were put under the bilateral cheek skin when two gold wire loop electrodes were carefully placed on the binocular surface. The ERG was recorded as full‐field ERG according to a standard protocol of the International Society for Clinical Electrophysiology of Vision (ISCEV). The retinal ERG a‐wave and b‐wave were recorded and averaged. The histopathological observation was assessed 12 weeks post‐injection. The eyes and organs of the mice were collected for paraffin pathological staining. The 5 µm sections were cut out and stained with hematoxylin‐eosin dye to visualize the structure and morphology of ocular and organ tissue.

### Statistics

Statistical analyses were performed with GraphPad Prism 8. The results are expressed as the means ± SEM. Statistical analysis for multiple comparisons was determined using an analysis of variance (ANOVA). The data from three independent experiments in which the variance was similar between the groups were analyzed using an unpaired and two‐tailored t‐test. *p*‐values of < 0.05 were considered statistically significant. All relevant data were available from the authors.

## Conflict of Interest

Outside the submitted work, Dr McAlinden has received consultancy fees/honorarium/travel support (past 36 months) from: Acufocus (Irvine, California, USA), Atia Vision (Campbell, California, USA), Bausch and Lomb (Bridgewater, New Jersey, USA), Bayer (Leverkusen, Germany), British Society of Refractive Surgery (Oxford, UK), BVI (Liège, Belgium), Coopervision (Pleasanton, California, USA), Cutting Edge (Labége, France), Hoya (Frankfurt, Germany), Knowledge Gate Group (Copenhagen, Denmark), Johnson & Johnson Surgical Vision (Santa Ana, California, USA), Keio University (Tokyo, Japan), Medevise Consulting SAS (Strasbourg, France), Ophtec BV (Groningen, The Netherlands), Portuguese Society of Ophthalmology (Coimbra, Portugal), ROHTO (Tokyo, Japan), Royal College of Ophthalmologists (London, UK), SightGlass vision (Menlo Park, California, USA), Science in Vision (Bend, Oregan, USA), Scope (Crawley, UK), SpyGlass (Aliso Viejo, California, USA), Sun Yat‐sen University (Guangzhou, China), Thea pharmaceuticals (Clemont‐Ferrand, France), Vold Vision (Arkansas, USA). Dr McAlinden developed the Quality of Vision (QoV) questionnaire and the Orthokeratology and Contact Lens Quality of Life Questionnaire (OCL‐QoL), and has a financial interest in these tools. He also consults on topics including Rasch analysis, questionnaires, statistical analyses, and clinical/surgical ophthalmology topics. Dr McAlinden is a co‐applicant on an awarded Welsh Government research grant related to diabetic eye disease (unpaid role), a council member of the British Society for Refractive Surgery (unpaid role), and a PROM advisor to the Royal College of Ophthalmologists (unpaid role). Dr McAlinden has undertaken paid peer reviews for Research Square (Durham, North Carolina, USA).

## Author Contributions

Z.Z., S.L., X.Y., Y.W., and Q.W. contributed equally to this work. Z.Z. and J.H. have conceptualized the work. Z.Z., S.L., Z.C., X.Y., and Y.W. have contributed to the methodology of the work. Z.Z. and Z.Z. looked after the software. Z.Z., Z.W., J.Z., and Q. W. have validated the work. Z.Z., S.L., Z.Z., and M.L. have performed the formal analysis. Z.Z., S.L., X.Y., Y.W., L.C., S.Z., and G.Z. did the investigation in the work. Q.W., J.H., and X.Z. provided the resources for the work. Z.Z., S.L., Z.C., Q.W., X.Y., Y.W., Z.W., J.Z., and X.C. did the data curation for the work. Z.Z. and S.L. wrote the original draft. H.L., Q.W., J.H., C.M., and X.Z. reviewed and edited the manuscript; Visualization, Z.Z. and S.L. did the visualization. Supervision, Q.W., and J.H. did the supervision of the work. Z.Z. and J.H. did the project administration, and X.Z. and J.H. took care of the funding acquisition for the work.

## Supporting information

Supporting Information

## Data Availability

The data that support the findings of this study are available from the corresponding author upon reasonable request.
