# Supplementary material for: Genome Editing VEGFA Prevents Corneal Neovascularization In Vivo
Source: Adv Sci (Weinh). 2024 Apr 6;11(25):2401710. doi: 10.1002/advs.202401710 (PMC11220714; doi:10.1002/advs.202401710)
Supplement: Supplementary file 1 — Supporting Information [file ADVS-11-2401710-s001.pdf]

ADVANCED SCIENCE
Open Access

Supporting Information

for *Adv. Sci.*, DOI 10.1002/advs.202401710

Genome Editing *VEGFA* Prevents Corneal Neovascularization In Vivo

Zhenhai Zeng, Siheng Li, Xiuhong Ye, Yiran Wang, Qinmei Wang, Zhongxing Chen, Ziqian Wang, Jun Zhang, Qing Wang, Lu Chen, Shuangzhe Zhang, Zhilin Zou, Meimin Lin, Xinyi Chen, Guoli Zhao, Colm McAlinden, Hetian Lei, Xingtao Zhou* and Jinhai Huang**

Supporting Information

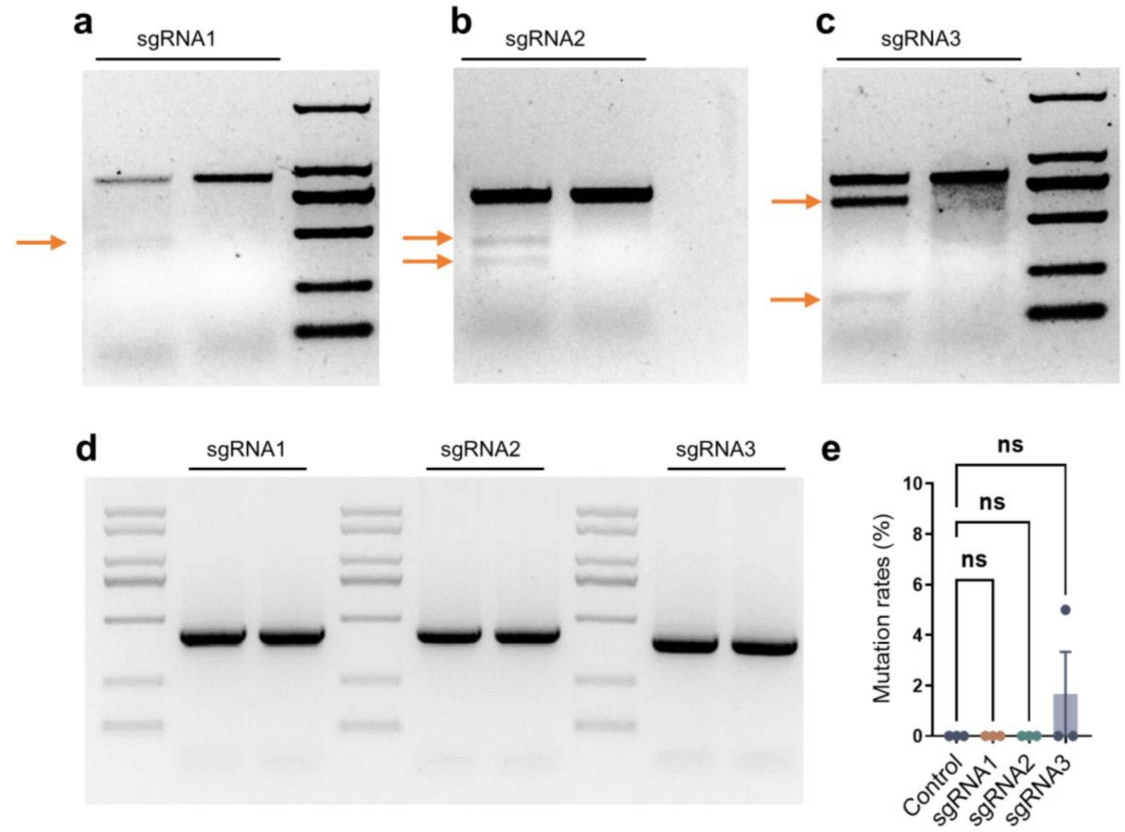

Supplementary Fig. 1 T7EI assay of on-target sites and off-target sites of the SpCas9/sgRNA1-3. **a-c.** Detection of the editing efficiency of *VEGFA* in HEK293T with a T7E1 assay. **d-e.** T7EI assay of the potential off-target sites of the SpCas9/sgRNA1-3.

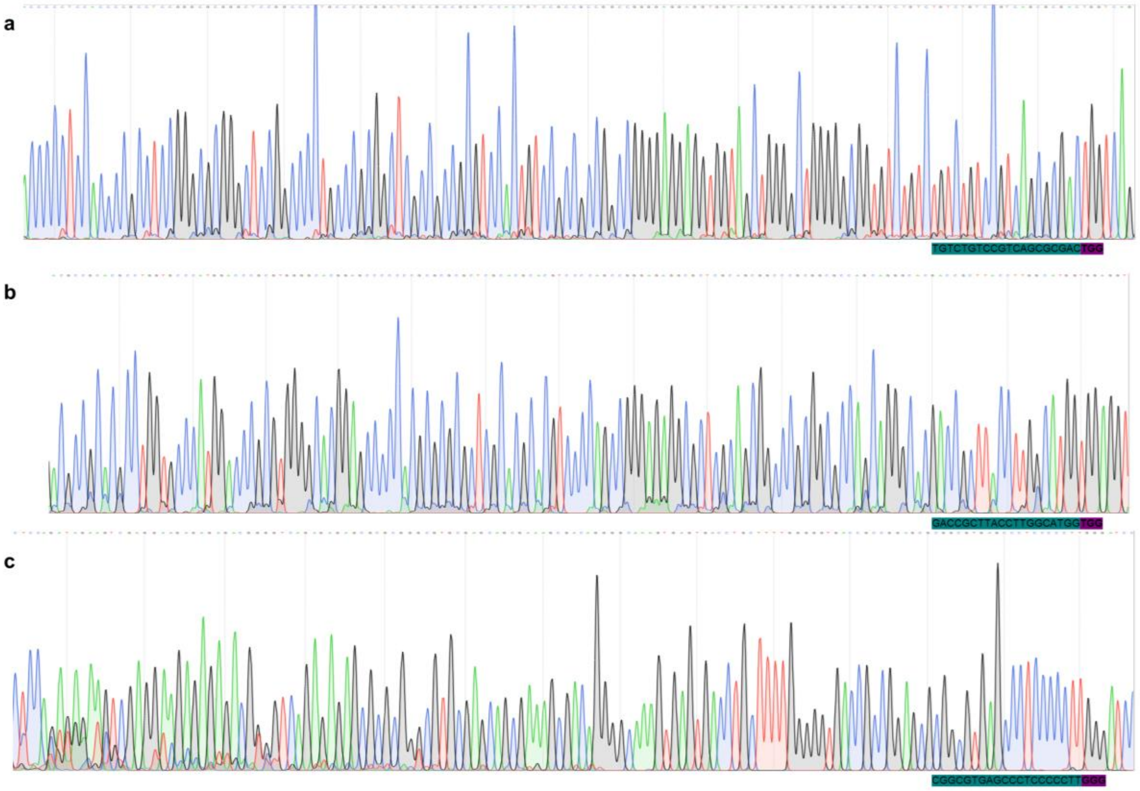

Supplementary Fig. 2 Sanger DNA sequencing of the 3 sgRNA for editing genomic *VEGFA*. a-c. Sanger DNA sequencing of the genomic loci around target sites. Green font indicates proto-spacer sequences, purple font indicates PAM sequences.

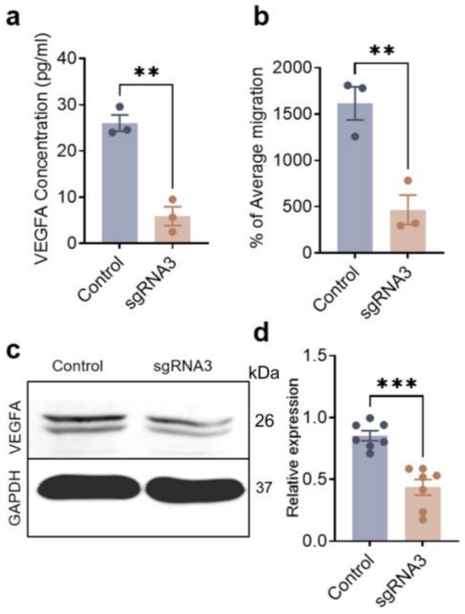

Supplementary Fig. 3 ELISA and WB assay were used to detect the expression level of VEGFA protein in RAW264.7 cell supernatant and cell lysate after transfection with CRISPR/spCas9 plasmids. **a.** ELISA detection of the expression level of VEGFA protein in the supernatant culture after transfected with SpCas9-sgRNA3 plasmids cultured for 48 hours. **b.** ELISA to detect the expression level of VEGFA protein in the RAW264.7 cell lysate after transfected with SpCas9-sgRNA3 plasmids cultured for 48 hours. **c-d.** WB assay and quantitative analysis of RAW264.7 cell lysate after transfection with SpCas9-sgRNA3 plasmids. Dots represent individual values and bars represent the mean ± SEM., n≥3.

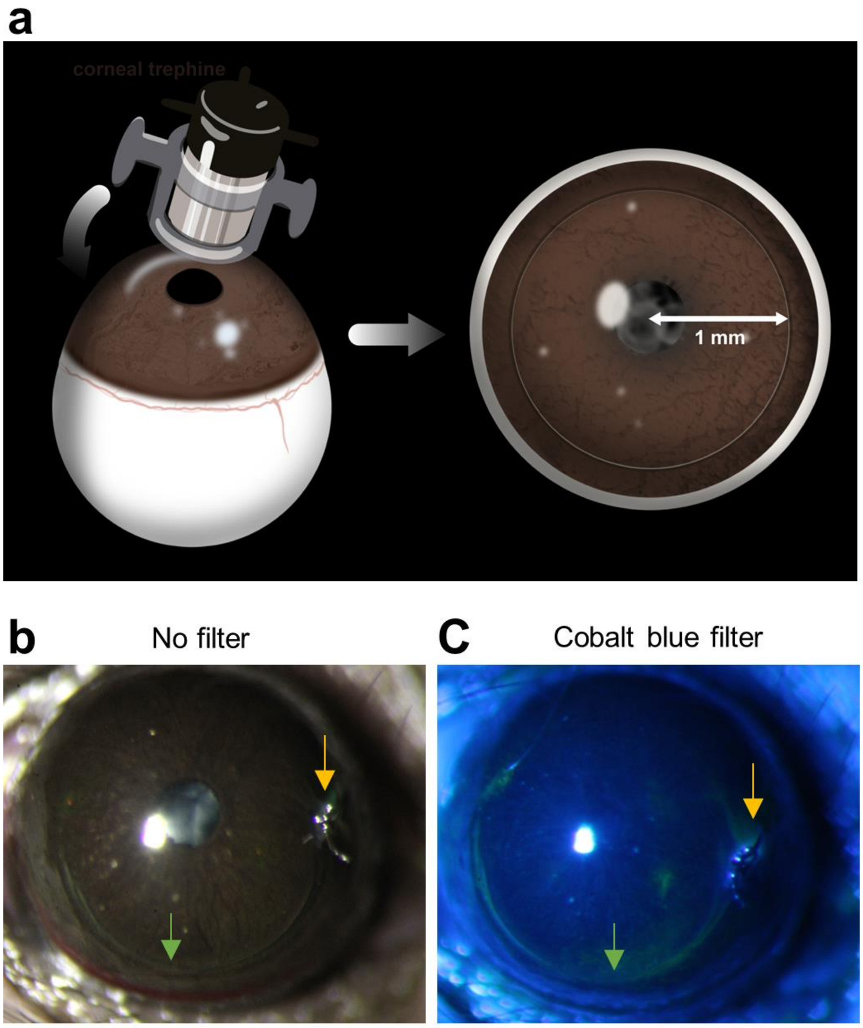

Supplementary Fig. 4 Anterior segment photography shows the position of corneal sutures A corneal trephine was utilized to demarcate a 2mm diameter circle centered around the pupil, upon which corneal neovascularization induced by sutures was performed.

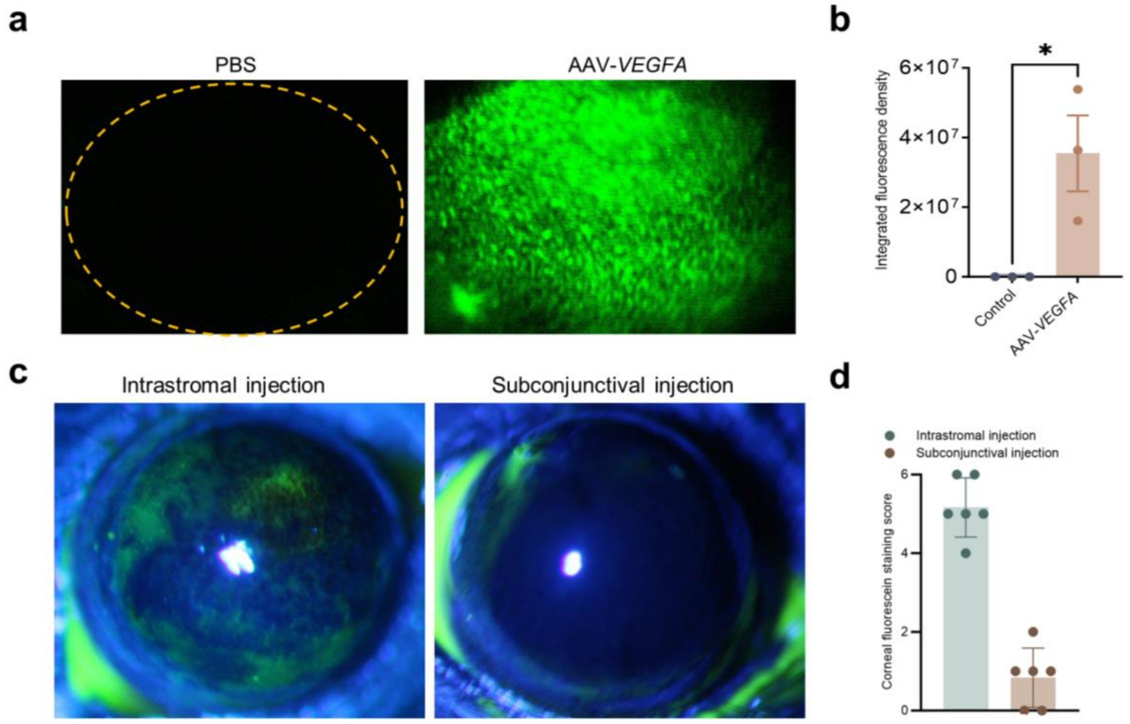

Supplementary Fig. 5 In vivo fluorescence photography observation of cornea to evaluate the infection range of dual AAV vectors of CRISPR/SpCas9-*VEGFA* system and comparative analysis of the intrastromal injection and subconjunctival injection. **a, b.** In vivo fluorescence photography and quantitative analysis to evaluate the expression of dual AAV vectors of CRISPR/SpCas9-*VEGFA* system in the cornea. **c.** Anterior segment photography of the intrastromal injection and subconjunctival injection. **d.** The corneal fluorescein staining score of the subconjunctival injection and intrastromal injection.

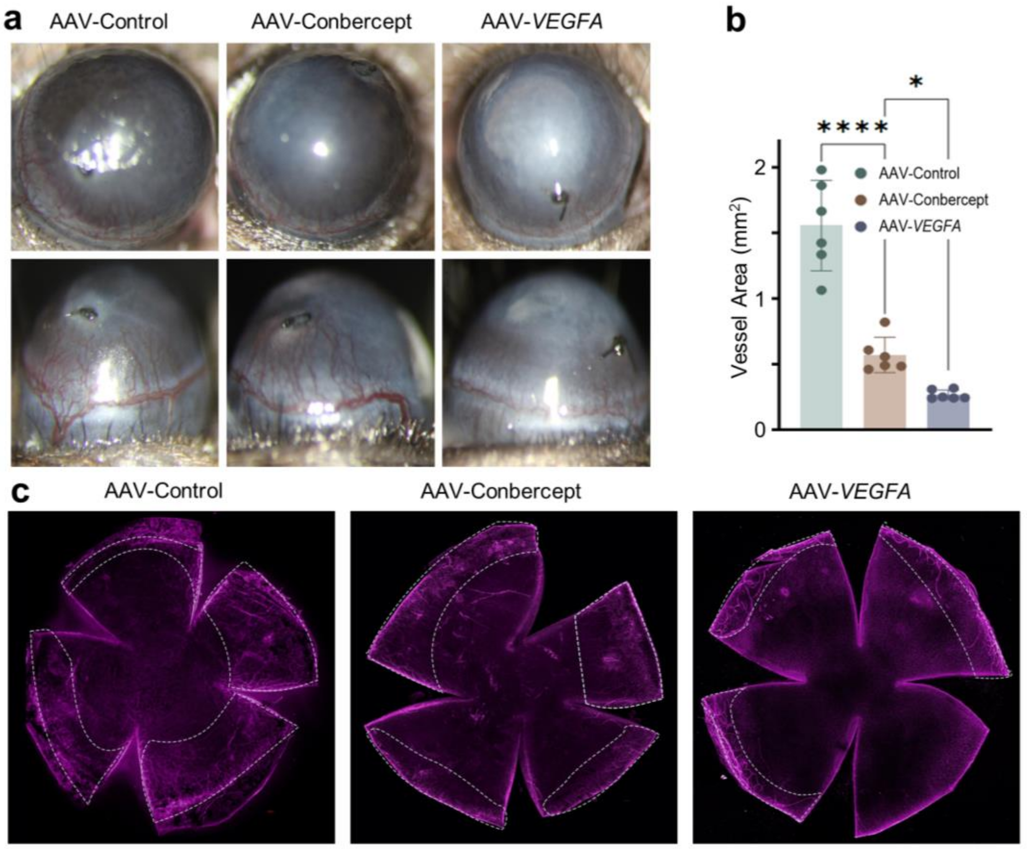

Supplementary Fig. 6 Comparative analysis of the effects of dual AAVs targeting *VEGFA* on CNV with Conbercept. **a.** Anterior segment photography demonstrated a superior inhibitory effect on corneal neovascularization compared to the Conbercept. **b.** Quantitative analysis of pathological corneal neovascularization. **c.** Whole cornea was immunofluorescence stained with CD31 antibodies, the white dashed boxes mark the area of corneal neovascularization.

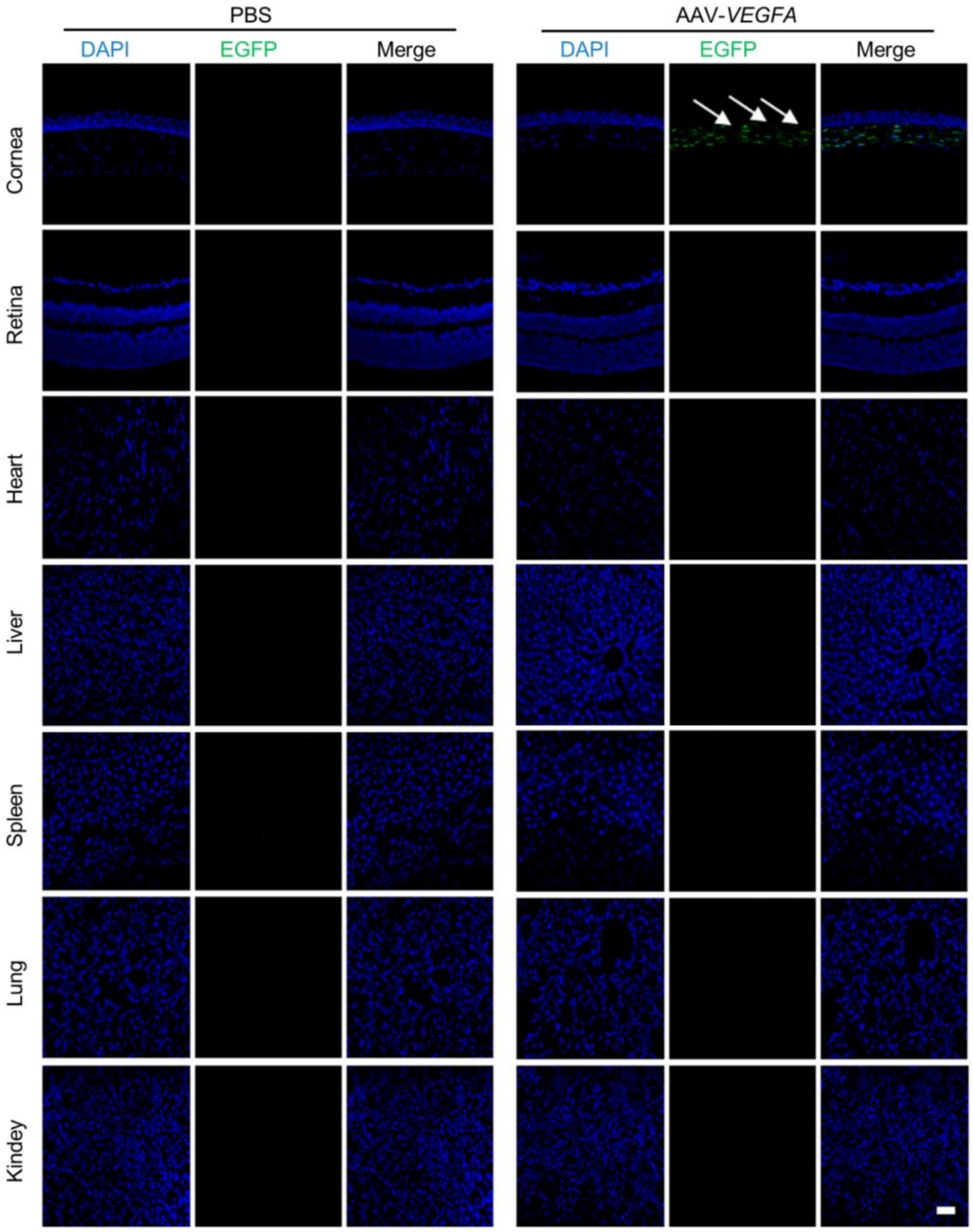

Supplementary Fig. 7. Cryosections of mouse cornea, retina, heart, liver, spleen, lung, and kidney after injection with the dual vectors of AAV-SpCas9 and AAV-sgRNA-*VEGFA*. Scale bar: 20μm.

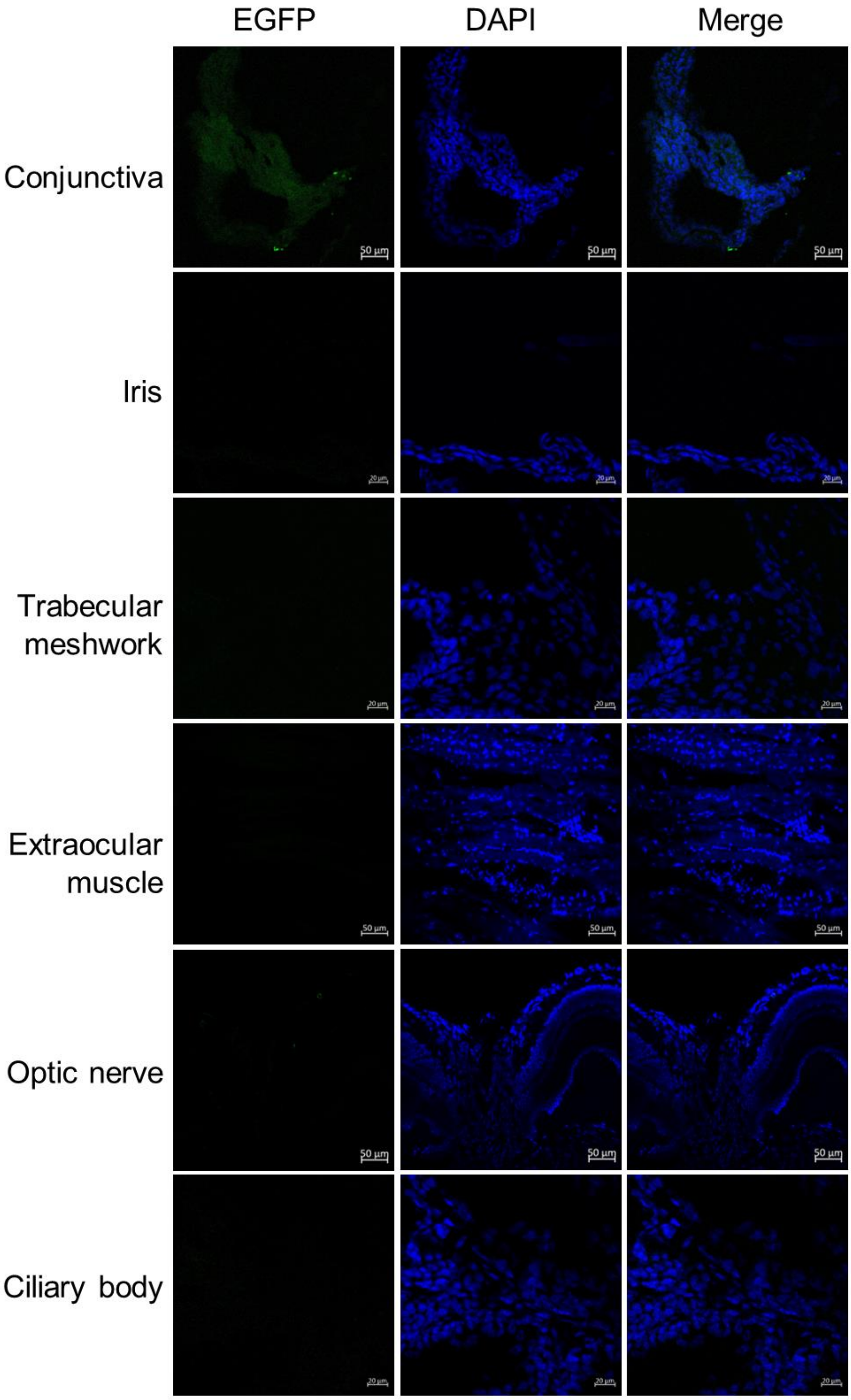

Supplementary Fig. 8 Biosafety of the dual vectors of AAV-SpCas9 and AAV-sgRNA-*VEGFA*. Cryosections of mouse conjunctiva, iris, trabecular meshwork, extraocular

muscle, optic nerve, and ciliary body after injection with the dual vectors of AAV-SpCas9 and AAV-sgRNA-*VEGFA*. Scale bar: 50μm.

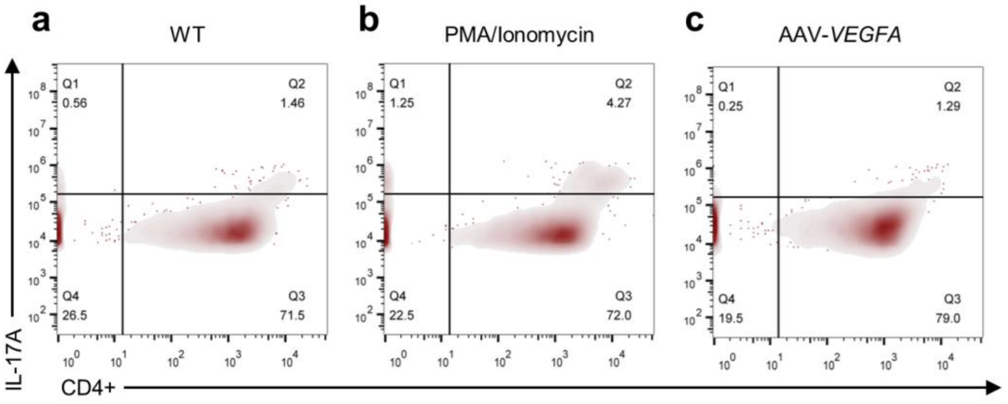

Supplementary Fig. 9 Flow cytometry showed the effect of the dual vectors of AAV-SpCas9 and AAV-sgRNA-*VEGFA* on activation of Th17 cells within the spleen. *a.* The quiescent state of the negative control. **b.** PMA/Ionomycin stimulation in the positive control. **c.** 7 days after subconjunctival injection of the dual AAVs targeting *VEGFA* system.
